# Factors associated with late antenatal care booking: population based observations from the 2007 Zambia demographic and health survey

**DOI:** 10.11604/pamj.2016.25.109.6873

**Published:** 2016-10-24

**Authors:** Nyambe Sinyange, Lungowe Sitali, Choolwe Jacobs, Patrick Musonda, Charles Michelo

**Affiliations:** 1 School of Public Health, School of Medicine, University of Zambia, Lusaka, Zambia; 2 Ministry of Health, Republic of Zambia

**Keywords:** Antenatal care, late, booking, factors, ZDHS

## Abstract

**Introduction:**

In spite of the extreme importance of an early antenatal care visit, more than 50% of Zambian pregnant women book for antenatal care late. We aimed to determine factors associated with late antenatal care booking in Zambia.

**Methods:**

Data stem from the 2007 Zambia Demographic and Health Survey where information on socio-demographic, social-economic, obstetrical characteristics and timing of the first antenatal visit were extracted on all women aged 15 to 49 years. A weighted survey analysis using STATA version 12 was applied. Firstly, we explored proportions of ANC booking at 0-3 months, 4-5 month and 6-9 months. Secondly, we investigated the association between predictor variables and late antenatal care booking using univariate and multivariate logistic regression.

**Results:**

Overall (n= 3979), the proportion of late ANC booking (booking between 4th to 9th month) was 81% disaggregated as 56% and 19% at 4 to 5 months and 6 to 9 months respectively. Women who wanted their last child later were more likely to book late than those with wanted pregnancies then (AOR: 1.35 95% CI 1.10-1.66). Women with higher education were 55% less likely to book for ANC late compared to women with no education (AOR: 0.45 95%CI: 0.27-0.74). Women aged 20-34 years were 30% more likely to book earlier than women younger than 20 years (AOR: 0.69 95% CI 0.50-0.97).

**Conclusion:**

We found high proportion of late ANC booking associated with presence of unplanned or unwanted pregnancies in this population. The concentration of this problem in lower or no education groups may be an illustration of existing inequalities which might further explain limitations in health promotion messages meant to mitigate this challenge. There is thus urgent need to re-pack health promotion message to specifically target this and related poor groups.

## Introduction

It is estimated that more than half of Zambian pregnant women have their first ANC (Antenatal Care) visit late [1] despite the extreme importance of an early ANC visit. The National Institute for Health Care Excellence (NICE) defines late booking as booking after 13 weeks and 6 days [2]. The Ministry of Health Zambia, in its latest HMIS primary procedures manual, defines late ANC booking as ANC booking later than 14 weeks of gestation [3]. On the other hand, early ANC booking preferably in the first trimester of pregnancy is very important as it enables early detecting and prevention any possible complications of pregnancy for the mother and the unborn child; thus, contributing to the reduction of perinatal morbidity and mortality [4]. Such practice will ensure good chances of safe delivery and delivery of healthy children [5]. Zambia has a high burden of maternal and neonatal deaths and coupled to this is a high burden of HIV and syphilis among pregnant women. According to the Ministry of Health (MoH), Zambia Antenatal Clinic Sentinel Surveillance Report 2008 to 2009, HIV infection rates amongst pregnant women were 16.4 per cent [6]. The Joint United Nations Program on HIV/AIDS (UNAIDS), Children and AIDS Fifth Stocktaking Report showed that 6800 infants are born each year from HIV infected women in Zambia [7]. This burden of disease could be reduced if pregnant women access antenatal care services early [8]. Other than HIV infection, syphilis infection among pregnant women has remained a significant challenge and although the Zambia Demographic and Health Survey (ZDHS) showed that syphilis prevalence amongst pregnant women was 4%, it is higher in selected sub-populations [1, 9].

To illustrate this point, the United Nations Children's Fund (UNICEF) report for Zambia highlighted that 591 mothers per 100,000 live births die while the neonatal mortality rate was 34 per 1,000 live births [10]. Some of this burden is arguably associated with preventable health challenges that could be detected during ANC clinics. Various studies across the world have shown that many factors can be associated with late ANC booking [11–18]. These include social-demographic factors, social-economic factors and biological factors. Other factors may be barriers faced by women in accessing health care and these barriers may also influence their decisions to attend ANC early. Evidence from studies done in the UK and Tanzania show that social-demographic factors such as not being married or not living with the husband, being of ethnic groups with a low social standing, are associated with late ANC booking [11–13]. Studies have also shown that social-economic factors such as the educational level of the woman, wealth level, employment status, exposure to media, lack of knowledge about importance of early ANC are associated with late ANC booking[14–18]. Biological factors like nulliparity, previous foetal loss and HIV status may be associated with late ANC booking as seen in studies done in Nigeria, Tanzania, Kenya, Zambia [19–22]. Women may experience barriers in accessing health such as long travel distances to health facilities, staff availability at the health facilities, staff attitudes at health facilities, problems getting money for treatment, having to take transport to the health facility and concerns about clinic booking procedures [23–26]. However information regarding this subject matter is limited in Zambia despite the opportunity that exits to analyse routinely collected information such as the ZDHS. In addition, further understanding on possible factors that may be associated with late ANC booking in Zambia is even more limited. We aimed to investigate factors that may be associated with late ANC booking in Zambia. The secondary objective was to determine the proportions of gestational age-specific late ANC bookings in the same population.

## Methods

### Study population

The study population was made up of women aged 15 to 49 that were interviewed during the ZDHS 2007. Only women who had a history of a live birth during the five years preceding the survey and attended antenatal care during their last pregnancy which resulted in a live birth were included in the study.

### ZDHS study design

The ZDHS was designed to provide estimates of population and health indicators at national and provincial levels. It consists of a stratified sample selected in two stages. The ZDHS 2007 obtained information on the extent to which women in Zambia receive care during pregnancy, during delivery and in the period after the baby is born. In the ZDHS 2007, women who had given birth in the five years preceding the survey were asked a number of questions about maternal care. For the last live birth in that period, mothers were asked if they had obtained antenatal care during the pregnancy. For women with two or more live births during the preceding five years, data obtained referred to the most recent birth. Detailed methods are reported in the 2007 ZDHS [1].

### Late ANC booking design

We conducted a cross-sectional study using secondary data given its large sample size that gives high powered estimates as well as high level of organisation in which the survey was conducted [9]. In this design, the information needed was extracted from the Women´s Questionnaire records and this included the women´s demographic characteristics, their full birth history (that is, history of a live birth during the five years preceding the ZDHS), and history of antenatal care for the most recent birth within a five-year period preceding the survey, if they could remember the number of months they had their first antenatal care visit to the health and this was recorded. Potential individual level background information available and judged to have potential to affect ANC utilization were also determined and extracted from the records. These variables included information on maternal age, social status and obstetrical factors at pregnancy represent maternal factors. Others were antenatal care visits and place of delivery to represent pre-delivery factors. These all included oonly predictor variables that were deemed important for the analysis and were thus subjected to further data cleaning process that included checking the extracted information about them for completeness. They include the following: age of the respondent, education level of respondent, number of years of education of respondent, residence, region, religion, total number of children ever born, marital status, last child wanted, knowledge of family planning , ever used family planning, media exposure to television, media exposure to newspaper, media exposure to radio, ever tested for HIV, wealth index, problems getting permission, problems getting money for treatment, problems with distance to the health facility, problems with having to take transport, problems with not wanting to go alone, concern that there might be no drugs, concern that there might not be a female health provider, concern that there might be no provider, age of the partner, education level of the partner and final decision maker for health care. The outcome variable was late antenatal care booking defined as attending first antenatal care after three months whereas early ANC booking was defined as attending first antenatal care visit for the first time in the first three months. The sample size included all the women who met the inclusion criteria.

### Data analysis

Data analysis was done using STATA software, version 12.0 SE (Stata Corporation, TX, USA). A weighted survey analysis was done due to the fact that this was survey data. Sampling weights used were the pweights; which denote that the inverse of the probability that the observation is included due to the sampling design and or non-response. Firstly descriptive statistics were done to determine the frequencies of late antenatal care booking. This included cross tabulation to determine the overall distribution of predictor variables by early or late ANC booking. This was followed by univariate logistic regression. Significance at univariate logistic regression was set at a p-value of 0.1 and a 95 per cent confidence interval. Variables that were found to be significant at univariate logistic regression were then fitted into the multiple logistic regression to control for confounding and to come up with the final model of predictor variables. For multiple logistic regression, significance was set at a p-value of 0.05 and 95 per cent confidence interval. Variables with the largest p-values were then removed one at a time until only significant variables were left in the final model. The analysed information was summarised using tables and graphs.

### Ethical considerations

Ethics approval (Reference number 2014-May-012) was obtained from the Excellence in Research Ethics and Science (ERES) converge while permission to obtain and use data from ZDHS 2007 was obtained from the Central Statistical Office (CSO). No information regarding names of participants was obtained. The data set was handled with confidentiality and only used for purposes of this study.

## Results

### Participation and distribution

In 2007, 3320 rural women and 4088 urban women were listed and overall response rate was 97 per cent. Of the de facto eligible and successfully interviewed population of all females aged 15 to 49, only the records of respondents that had completed the interview (n=3979), including answering the information on ANC attendance were included in the final analysis. Of the 3979 women 67% (2666) were from rural areas while 33% (1313) were from urban areas. The mean age overall was 28 (±7) years but was 29 (±7) years for rural and 27 (±7) years for urban. In general, the majority (72%) of women were aged between 20 and 34 years, 79% of them married and 60% only attained primary education. The frequency distribution of predictor variables by timing of ANC booking is summarised in Table 1.

**Table 1:** Frequency distribution of selected predictor variables of late ANC booking by timing of ANC booking among women aged 15-49 years in Zambia, stratified by rural and urban

Characteristic	Overall			Rural			Urban		
	*Early*	*Late*		Early-ANC booking	Late-ANC booking	P-value (chi²)	Early-ANC booking	Late-ANC booking	P-value (chi²)
	*Number (%)*	*Number (%)*	*P-value*	*Number (%)*	*Number (%)*		*Number (%)*	*Number (%)*	
**Age group (years) of respondent**									
<20	47 (14)	283 (86)	0.04	33 (16)	179 (84)	0.47	14 (12)	104 (88)	0.04
20-34	584 (20)	2267 (80)		358 (19)	1505 (81)		226 (23)	762 (77)	
35-49	150 (19)	653 (81)		108 (18)	486 (82)		41 (20)	167 (80)	
**Educational level of respondent**									
No education	103 (21)	397 (79)	<0.01	97 (21)	356 (79)	<0.01	6 (13)	41 (87)	<0.01
Primary	430 (18)	2001 (82)		331 (18)	1503 (82)		99 (17)	498 (83)	
Secondary	200 (22)	728 (78)		65 (17)	310 (83)		135 (24)	418 (76)	
Higher	48 (38)	76 (62)		6 (75)	2 (25)		42 (36)	74 (63)	
**Last child wanted**									
Wanted then	464 (22)	1657 (78)	0.01	306 (20)	1185 (80)	0.03	158 (25)	472 (75)	0.02
Wanted later	184 (17)	908 (83)		115 (17)	577 (83)		69 (17)	331 (82)	
Wanted no more	133 (17)	638 (83)		78 (16)	409 (84)		55 (19)	229 (81)	
**Problems getting money for treatment**									
Yes	235 (16)	1156 (83)	<0.01	186 (17)	877 (83)	0.25	49 (15)	279 (85)	<0.01
No	547 (21)	2047 (79)		314 (19)	1296 (81)		233 (24)	751 (76)	
**Concern that there might not be drugs**									
Yes	457 (21)	1756 (79)	0.13	326 (20)	1292 (80)	0.04	131 (22)	464 (78)	0.68
No	324 (18)	1447 (82)		173 (16)	880 (84)		151 (21)	567 (79)	

### ANC Attendance

**Figure 1:**
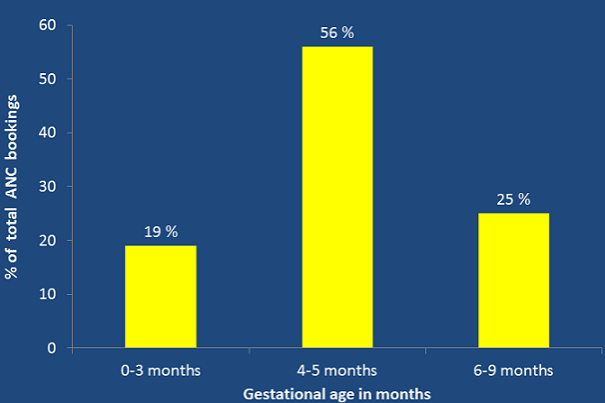
Proportions of ANC booking in month’s categories in Zambia

**Figure 2:**
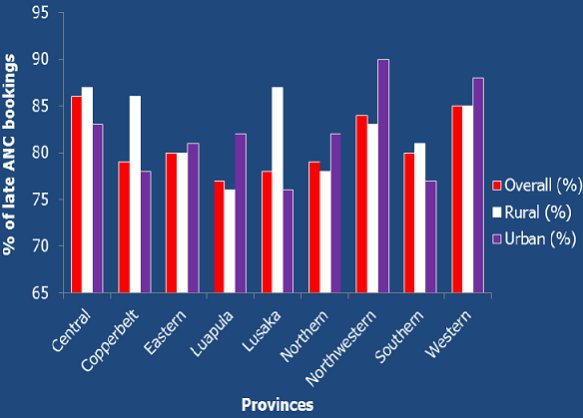
Proportions of Late ANC booking by provinces of Zambia

The proportion of late ANC attendance (being first ANC visit at 4 to 9 months) was 81% (95% CI 80%, 82%); whereas only 19% (19%, 95% CI 18%, 21%) of the women had their first antenatal care visit within the first three months of pregnancy (0-3 months). Of those who had the first ANC booking after three months, 55% had their first antenatal care visit at 4 to 5 months gestation and only 26% of the women attended their first ANC visit between 6 and 9 months (see Figure 1). There were observed rural urban differentials. Of the women in the rural areas, 81% (n=2167) had their first ANC visit after three months of gestation while in the urban area, it was 79%. In all the provinces, more than 50 percent of women attended their first ANC visit late (see Figure 2). Further, Figure 2 shows that there was a high proportion of late ANC booking in the urban areas from Eastern, Luapula, Northern, North Western, and Western provinces whereas there was a high proportion of late ANC booking in the rural areas from Central, Copperbelt, and Lusaka provinces.

### Determinants of Late ANC attendance

Univariate logistic regression analysis of social-demographic factors showed that women in the age group 20 to 34 years were associated with a decreased (p-value 0.009, CI 0.478-0.880, OR 0.653) chance of late ANC booking compared to women younger than 20 years. The respondents who had expressed that they would have loved to have delayed having a child when they conceived had a higher likelihood of booking late for ANC (OR 1.37 95%CI 1.13-1.69). This was also true for those who did not want to have a child anymore (OR 1.34 95% CI 1.08-1.67) (see Table 2). Higher educational level on the other hand was associated with a significant decreased likelihood of late ANC booking compared to those with no education (OR 0.42, 95%CI 0.26-0.69) and this was most pronounced in groups exposed to television daily (OR 0.69 95%CI 0.54-0.89) and newspapers (OR 0.63 95%CI 0.41-0.98). In addition, women with more than five children ever born was associated with an increased chance of late ANC booking compared to those with one child ever born (OR 1.77, 95%CI 1.23-2.54) and this was most pronounced in urban than rural populations.

**Table 2:** Final model for predictor variables for late ANC booking among women aged 15-49 years in Zambia

Predictors of late ANC booking	Overall		Rural		Urban	
	Prevalence (%)	AOR (95% CI)+	AOR (95% CI)+	P-value+	AOR (95% CI)+	P-value+
**Last child wanted**						
Wanted then	78	1	1	1	1	1
Wanted later	83	1.35 (1.10-1.66)	1.28 (1.00-1.65)	0.05	1.44 (0.99-2.09)	0.05
Wanted no more	83	1.27 (1.02-1.59)	1.31 (0.99-1.74)	0.05	1.17 (0.79-1.72)	0.42
**Age of respondent (years)**						
<20	86	1	1	1	1	1
20-34	80	0.69 (0.50-0.97)	0.81 (0.53-1.24)	0.34	0.53 (0.31-0.88)	0.01
35-49	81	0.75 (0.53-1.06)	0.86 (0.56-1.33)	0.50	0.58 (0.32-1.09)	0.089
**Educational level of respondent**						
No education	79	1				
Primary	82	1.19 (0.93-1.54)	1.24 (0.94-1.63)	0.12	0.78 (0.29-2.13)	0.62
Secondary	78	0.92 (0.68-1.22)	1.24 (0.86-1.79)	0.24	0.50 (0.18-1.36)	0.17
Higher	62	0.45 (0.27-0.74)	0.08 (0.08-0.38)	<0.01	0.34 (0.12-0.96)	0.04
**Problems getting money for treatment**						
Big problem	83	1				
Not a big problem	79	0.76 (0.63-0.92)	0.83(0.65-1.06)	0.13	0.62 (0.47-0.82)	<0.01
**Concern that there might be no drugs**						
Big problem	79	1				
Not a big problem	82	1.26 (1.03-1.53)	1.34 (1.05-1.70)	0.02	1.17 (0.84-1.62)	0.35
**Knowledge of family planning methods**						
No	68	1	1	1	1	1
Yes	81	2.16 (1.16-4.03)	2.39 (1.28-4.45)	<0.01	n/a	n/a

**AOR=** Adjusted Odds Ratio **OR=** Odds Ratio **CI=** Confidence Interval

+ Logistic regression

### Core Determinants of late ANC booking

In multivariate logistic regression analysis, core determinants for late ANC booking found were unplanned pregnancy, satisfied parity, as well as rural older women. Women who wanted their last child to have come later were associated with an increased (AOR 1.35 95% CI 1.01-1.66,) chance of late ANC booking compared to their comparison group. Similarly, those who did not want to have any more children had an increased chance to delay ANC booking (AOR 1.27, 95%CI 1.02-1.59).Young women in age group 20-34 tended to book early (AOR 0.69 95%CI 0.50-0.96) and this was most pronounced in urban groups with higher education (AOR 0.45 95%CI 0.27-0.74).

## Discussion

A high proportion of late ANC booking in lower educated rural groups with unplanned or satisfied parity and women younger than 20 years were the core drivers observed in this population. Similar results in studies elsewhere also showed that that women aged 15 to 19 years were more likely to book for ANC late [27, 28]. The reasons for this are unclear but it is reasonable to think that young women aged 15 to 19 years are teenagers and may be more likely be unmarried or may not know or understand the importance of booking for ANC early. Women older than 19 years on the other hand may be more likely to be married and starting family for the first time, and might be anxious about their safety as well as their babies hence the ease with which they book and attend ANC [28]. Some of this might be related to availability and affinity of health promotion messages regarding child and maternal survival. In the present it was thus not surprising to find that women exposed to the media tended to book early suggesting that they may have heard health promotional messages that modified positively their understanding on the role of ANCs in both child and maternal survival [16, 29].

It looks reasonable that unplanned pregnancies may not receive much attention regarding early ANC booking as shown in this study. Various barriers to early ANC booking may surround unplanned pregnancy and may include shame due to conception at old age, poor family spacing or delayed diagnosis of pregnancy. Results similar to the findings in this study were seen in a study done in the Copperbelt province of Zambia which showed that women with unplanned pregnancy were 4.4 times more likely to book for ANC late compared to those with planned pregnancies [21]. A survey done in Tanzania similarly showed that women with unplanned pregnancies were two times more likely to book for ANC late compared to women with planned pregnancies [30]. The question which arises is whether the women know about any family planning methods. If they know about family planning methods, do they have access them. Do the health promotion messages cater for those with unplanned pregnancies to encourage them to book early? While it sounds logical that women with knowledge about family may book for ANC early, the results from this study did not support this reasoning. What this may be telling us is that women may be aware of the family planning methods but my fail to either access them or use them correctly.

In this study, we observed that women with higher education are more likely to book for ANC earlier than women with no education. This is supported by studies done in Nigeria, New Zealand and a systematic review of literature from African countries similarly showed that women with higher education were more likely to book for ANC early [4, 14, 16, 31, 32].This may be attributed to differencees in the levels of understanding the importance of early ANC booking among the different levels of education of women [27, 28]. This probably explains why the variables media exposure to television and newspaper were significant at univariate logistic regression analysis and seem to be confounding at multiple logistic regression analysis. This suggests that women with higher education are more likely to read newspapers and watch television more frequently than less educated or not educated women.

Among barriers women faced in accessing health care, it was expected that distance to health facilities would be significant, but contrary to that, distance was insignificant at both univariate and adjusted logistic regression analysis. What appeared to be significant were women who had problems in getting money for treatment and women who were concerned that there might be no drugs at the facility [31]. Women who had no problems getting money for treatment were 36 percent less likely to book for ANC late. Those women who thought non-availability of drugs at the health facility was not a big problem were 1.26 times more likely to book late. This may mean that they drugs availability did not worry them or influence their decision to book for ANC early. These grey areas could be addressed by a mixed-method study so that the qualitative component could be addressed to highlight specific issues the women who book late for ANC have.

One limitation in the use of the survey data reported here was that the latest ZDHS data were only available for 2007 at the time of the study and that is about seven years ago suggesting that this picture may have changed. However and because this study was not linked to evaluations of associated maternal survival strategies, it is difficult to estimate the effect of this limitation. Other limitations were that some variables of interest like education of the partner and age of the partner had significantly missing data and could not be included in the analysis and certain questions could only be answered using the qualitative method approach and were thus not included in this analysis. The 2007 ZDHS only asked women with previous live birth in the five years preceding the survey questions about ANC and this may be a source of bias because those women without live births may be the ones with big challenges in booking for ANC early. Despite these limitations, the observed results were from a representative high-powered population based sample of women in Zambia and we are confident that they still point to significant problems associated with late ANC booking which are difficult to ignore.

## Conclusion

In this study, we found that late ANC booking is still a problem in Zambia, suggesting that a large proportion of pregnant women in Zambia miss the opportunity to have early detection and prevention any possible complications of pregnancy and the unborn child. These observations further show that this challenge is concentrated in women younger than 20 years of age, women with unwanted pregnancies and women with no education. The concentration of this problem in lower or no education groups may be an illustration of existing inequalities which might further explain past and present limitations in health promotion messages meant to mitigate this challenge. This could also have strategic hindrances in planned interventions aimed at enhancing both maternal and child survival. There is thus urgent need to re-pack health promotion message to specifically target these population sub-groups. In addition we thus recommend that access to family planning methods as well as benefits early ANC booking and attendance be made as a right. To support this, the health promotion packages that encourage pregnant women to book for ANC early must thus be repackaged to suit differential background characteristics that account for their differences in education status, age and parity as observed in this study. This is critical as these factors may have been missed in past maternal survival strategies in this population. If we look at this as a right for the women, an obligation for policy makers and a responsibility for primary care workers, we may save many lives, and consequently save millions of dollars currently incurred in fire fighting.

### What is known about this topic

- More than half of Zambian pregnant women have their first ANC visit late;
- There is need for early ANC booking preferable before 14 weeks gestation in order to increase the chances of safe delivery and delivery of health infants;
- Education, marital status, distance to health facilities, age, ethnicity, parity, unplanned pregnancies, lack of privacy at health institutions, misconceptions about ANC and cultural beliefs have been associated with late ANC booking.

### What this study adds

- This study utilised secondary data from the 2007 ZDHS, a high sample with 97 percent response rate, to determine the factors associated with late ANC booking;
- The key drivers of late ANC booking in this study were lower educated rural groups with unplanned or satisfied parity and women younger than 20 years.
